# CLEC14A correlates with neutrophil infiltration in hepatocellular carcinoma and mediates neutrophil recruitment across liver endothelial cells

**DOI:** 10.1002/path.70077

**Published:** 2026-06-01

**Authors:** Joanne M O'Rourke, Dean Kavanagh, Ayla O'Keeffe, Kylie Savoye, Kaiyi Yang, Daniel A Patten, Catherine E Willoughby, Josep M Llovet, Georgiana Neag, Helen L Reeves, Peter W Hewett, Neena Kalia, Tahir Shah, Yuk Ting Ma, Kelly Hunter, Joe Flint, Jack L McMurray, Owen Cain, Fabian Spill, Derek A Mann, Amy J Naylor, Victoria Heath, Christopher J Weston, Roy Bicknell, Shishir Shetty

**Affiliations:** ^1^ Centre for Liver and Gastrointestinal Research, Department of Immunology and Immunotherapy, College of Medicine and Health University of Birmingham Birmingham UK; ^2^ Microcirculation Research Group, Department of Cardiovascular Sciences, College of Medicine and Health University of Birmingham Birmingham UK; ^3^ School of Mathematics, University of Birmingham Birmingham UK; ^4^ Mount Sinai Liver Cancer Program, Division of Liver Diseases, Department of Hematology/Oncology, Department of Medicine Tisch Cancer Institute, Icahn School of Medicine at Mount Sinai New York NY USA; ^5^ Liver Cancer Translational Research Laboratory, Institut d'Investigacions Biomèdiques August Pi i Sunyer (IDIBAPS), Hospital Clínic Universitat de Barcelona Barcelona Spain; ^6^ Institució Catalana de Recerca i Estudis Avançats (ICREA) Barcelona Spain; ^7^ Department of Inflammation and Ageing, College of Medicine and Health University of Birmingham Birmingham UK; ^8^ Newcastle University Translational and Clinical Research Institute, The Medical School Newcastle‐upon‐Tyne UK; ^9^ Department of Cardiovascular Sciences, College of Medicine and Health University of Birmingham Birmingham UK; ^10^ Liver Unit Queen Elizabeth Hospital Birmingham UK; ^11^ Department of Immunology and Immunotherapy University of Birmingham Birmingham UK; ^12^ Birmingham Tissue Analytics, College of Medicine and Health University of Birmingham Birmingham UK; ^13^ Department of Pathology Queen Elizabeth Hospital Birmingham Birmingham UK; ^14^ Newcastle Fibrosis Research Group, Biosciences Institute, Faculty of Medical Sciences Newcastle University Newcastle‐upon‐Tyne UK; ^15^ National Institute for Health Research Birmingham Biomedical Research Centre, University of Birmingham Birmingham UK

**Keywords:** CLEC14A, neutrophil, hepatocellular carcinoma, tumour endothelium, adhesion

## Abstract

Hepatocellular carcinoma (HCC) is a leading cause of cancer‐related deaths globally, and cases are predicted to rise dramatically over the next few years. Overcoming the immune microenvironment in HCC remains a challenge, and innate immune populations such as tumour‐associated neutrophils can potentially impair the success of immunotherapy. Elucidating the mechanisms of neutrophil recruitment across liver endothelium could lead to new approaches to boost the efficacy of immunotherapy. CLEC14A is an endothelium‐specific receptor regulating sprouting angiogenesis, upregulated in low shear environments. We found CLEC14A to be highly expressed in both HCC vessels and on vasculature during acute liver injury, leading us to hypothesise that CLEC14A may regulate neutrophil recruitment to the liver and HCC. We found that CLEC14A positively correlated with a neutrophil signature and infiltration in public datasets and surgically resected tissue from HCC. Spatial transcriptomics (ST) of CLEC14A^high^‐ and CLEC14A^low^‐expressing tumours confirmed upregulation of myeloperoxidase in CLEC14A^high^ tumours and correlation with other shear‐dependent markers but a negative correlation with vascular endothelial growth factor A. To build on our correlation studies, we explored the role of CLEC14A in neutrophil recruitment across liver endothelium. We undertook *in vitro* recruitment studies with flow‐based human liver endothelial assays and *in vivo* models of neutrophil recruitment. Using siRNA knockdown of *CLEC14A* and specific blocking antibodies to CLEC14A, we found that *CLEC14A* knockdown blocked the firm adhesion of neutrophils to liver endothelium, but this was independent of its interaction with its known ligand MMRN2. Finally, we confirmed that *Clec14a* deficiency led to a significant reduction in neutrophil recruitment across the sinusoids in an ischaemia‐reperfusion liver injury model. We unveil a link between the angiogenic receptor CLEC14A and neutrophil recruitment. Targeting of CLEC14A on tumour endothelium is potentially a new approach to regulating neutrophil infiltration in HCC. © 2026 The Author(s). *The Journal of Pathology* published by John Wiley & Sons Ltd on behalf of The Pathological Society of Great Britain and Ireland.

## Introduction

Hepatocellular carcinoma (HCC) is a leading cause of global cancer deaths, and cases continues to rise worldwide [1]. It is estimated that globally one million people will be affected by primary liver cancer in 2025. Recent breakthroughs in therapy for this lethal tumour include the combination of immunotherapy with anti‐vascular endothelial growth factor (VEGF) therapy, highlighting the potential of targeting vasculature to regulate the immune microenvironment [2, 3]. Finding new vascular targets independent of VEGF to shape the immune microenvironment of HCC could have a major impact, as currently only 30% of patients with advanced HCC respond to therapy.

Recent studies have implicated neutrophils as active players in the tumour microenvironment (TME), demonstrating that they can play a critical role in the cross‐talk with the adaptive immune system and mediate anti‐tumour T‐cell responses in preclinical solid organ tumour models, including HCC [4, 5]. Identifying factors that regulate neutrophil recruitment to the TME of HCC could have important therapeutic implications.

Circulating neutrophils are directed to sites of damage by their interaction with endothelial cells lining the vasculature, making this a critical initiating event in neutrophil responses [6]. In the liver, leukocyte recruitment occurs within low‐flow channels of the hepatic sinusoids, which are lined by a phenotypically distinct endothelium formed by liver sinusoidal endothelial cells (LSECs) [7]. Tumour vascular beds are also characterised by low shear flow and potentially share recruitment characteristics with the liver sinusoidal channels. With this in mind, we focused on the C‐type lectin family member CLEC14A, which has previously been shown to be significantly upregulated on tumour endothelium and its expression is shear dependent, with low shear promoting endothelial expression [8].

## Materials and methods

For additional detailed description of some methods, see the Supplementary materials and methods.

### Ethics approval

Explant human liver tissue was collected from patients undergoing liver transplantation at the Queen Elizabeth Hospital Birmingham under ethical study numbers 06/Q2702/61, 18/WA/0214, and 18/LO/0102. Normal liver tissue was obtained from rejected organ donors deemed unsuitable for transplantation under ethical study numbers 06/Q2702/61 and 18/WA/0214. Peripheral blood samples were taken from healthy volunteers under ethical study number 18/WA/0214. Archival HCC surgical resection specimens were obtained under ethics 25/NW/0013 from the Human Biomaterials Resource Centre. Biopsy samples were collected under IRAS: 266624 and the REC approval reference 19/NE/0251.

All animal experiments were performed under a project licence issued by the UK Home Office and in accordance with the Animals in Scientific Procedures Act 1986.

### Histological assessment of tissue

Immunohistochemistry of CLEC14A of paraffin‐embedded sections was performed as previously described [9] using primary antibody CLEC14A polyclonal antibody (Bio‐Techne, Abingdon, Oxfordshire, UK) and secondary antibody anti‐sheep HRP (Bio‐Techne). For a cohort of HCC samples, matched serial sections were taken, followed by immunofluorescent staining for CD15 with primary antibody mouse anti‐human (Clone MC480, CST, Leiden, South Holland, The Netherlands) and secondary goat anti‐mouse Alexa Fluor (Invitrogen, Paisley, Renfrewshire, UK). Slides were imaged using a Zeiss Axio slide scanner (Zeiss, Cambridge, Cambridgeshire, UK) and visualised in Zen imaging software (Zeiss).

### Primary human LSEC isolation

LSECs were isolated from ∼30‐g slices of explanted or surgically resected human liver as previously described [10]. LSECs were maintained in human endothelial serum‐free medium (Gibco, Paisley, Renfrewshire, UK) supplemented with 10% human serum (TCS Biosciences, Buckingham, Buckinghamshire, UK), 1% PSG (Gibco), 10 ng/ml VEGF (Peprotech, Paisley, Renfrewshire, UK), and 10 ng/ml HGF (Peprotech).

### Western blotting

Whole‐cell (WC) protein lysates were performed as previously described [11]. Primary antibody (0.1 μg/ml CLEC14A polyclonal antibody, Bio‐Techne) was incubated overnight at 4 °C. The membranes were then incubated for 1 h at room temperature with anti‐sheep IgG‐conjugated with HRP (0.2 μg/ml, Bio‐Techne). HRP was detected using enhanced chemiluminescence (ECL) using Amersham ECL western blotting detection reagent (Cytiva/GE Healthcare, Little Chalfont, Buckinghamshire, UK) following the manufacturer's protocol and imaged using a LI‐COR Odyssey imager (LI‐COR Biotechnology, Cambridge, Cambridgeshire, UK).

Membranes were stripped with Restore Western Stripping Buffer (Thermo Fisher Scientific, Paisley, Renfrewshire, UK) for 10 min before repeating blocking and incubation steps above to probe for Multi‐merin‐2 (0.2 μg/ml, Abcam, Cambridge, Cambridgeshire, UK) or β‐actin (1 μg/ml, Merck, Gillingham, Dorset, UK). After washing, membranes were incubated with HRP‐conjugated anti‐mouse IgG antibody (1:5,000 Agilent, Stockport, Greater Manchester, UK) or HRP‐conjugated anti‐rabbit IgG antibody (0.2 μg/ml, Abcam) for 1 h.

### Real‐time quantitative PCR (RT‐qPCR)

Analysis of mRNA expression was performed using TaqMan Gene expression assays with 6 carboxyfluorescein‐labelled probes (Applied Biosystems, Paisley, Renfrewshire, UK). GAPDH was used as the housekeeping gene. The cycle threshold value (Ct) for the gene of interest was normalised against the housekeeping gene, and relative expression was calculated using the 2^−ΔΔCt^ method [12]. For the reaction, Taqman 2× master mix (Applied Biosystems) was used at a final concentration of ×1 together with 20× assay mix (Applied Biosystems) at ×0.5, 100 μg/ml of cDNA, in a total volume of 10 μl.

### RNA interference

Small interfering RNA (siRNA) targeting *CLEC14A* were purchased from Thermo Fisher Scientific (D1 – Assay ID 129879 and D2‐ Assay ID 129880). The sequences were D1‐GAACAAGACAATTCAGTAA and D2‐CAATCAGGGTCGACGAGAA). OptiMEM (800 μl) (Gibco) was added. For transfection, 1 μl of 20 μm siRNA was added to 167.5 μl of OptiMEM, and 3 μl of RNAiMAX lipofectamine (Invitrogen) was mixed with 27 μl OptiMEM. Both mixtures were kept at room temperature for 10 min. The 30‐μl mixture, with lipofectamine, was then added to the siRNA and subsequently added to the cells. No antibiotics were used. After 48 h, assays were performed, and knockdown was confirmed using western blotting. For use in flow adhesion assays, LSECs were seeded in rat tail collagen‐coated Ibidi μ‐slides (Thistle Scientific, Rugby, Warwickshire, UK), and siRNA knockdown (as described above) was performed *in situ*.

### LSEC exposure to hypoxia

Confluent LSECs in a 6‐well plate were cultured in a standard incubator in normoxic conditions or a hypoxic chamber (RS Mini Galaxy incubator, Wolf Laboratories, York, Yorkshire, UK) for 24 h at 2% O_2_.

### Isolation of human neutrophils

Histopaque (HP) 1199 and 1077 (Sigma, Gillingham, Dorset, UK) were warmed to room temperature for 30 min. Next, 2.5 ml of HP 1119 was added to a 15‐ml tube, and 2.5 ml of HP 1077 was carefully pipetted on top. Blood (5 ml) was added to the surface and then centrifuged at 500 × *g* for 45 min. Peripheral blood mononuclear cells (PBMCs) were removed first to minimise contamination, followed by neutrophils. PBS (10 ml) without Ca/Mg (Gibco) was added to the neutrophils. Neutrophils were centrifuged at 500 × *g* for 5 min, resuspended to 1 ml, and cells were counted and used immediately for assays.

### Isolation of human lymphocytes

PBMCs were isolated from whole blood using Lympholyte cell separation medium (Cedarlane, Burlington, Ontario, Canada) and centrifugation was performed at 800 × *g* for 20 min without brake. To isolate lymphocytes, PBMCs were resuspended in RPMI medium (Gibco) and placed in a tissue culture flask for 1 h to allow monocyte adhesion. The non‐adherent cell suspension was then harvested, centrifuged, and resuspended.

### Matrigel assays

Matrigel was thawed overnight on ice in a cold room and 70 μl added to each well. After 5 min in the incubator, 160,000 LSECs were added to each well and placed in a Cell IQ (CM Technologies, Tampere, Finland) and images captured hourly for 24 h. Images were analysed using the ImageJ ‘Angiogenesis Analyzer’ plugin https:imagej.net/ij/index.html, and the number of tubes per image was recorded.

### Leukocyte binding of recombinant CLEC14A

Neutrophils or lymphocytes were incubated with CLEC14A‐ECD‐Fc (R&D Systems, Abingdon, Oxfordshire, UK) or human Fc (Sigma) alone, which had been fluorescently labelled with Invitrogen Zenon human IgG labelling kit (Invitrogen). CLEC14A mutants were generated by site‐directed mutagenesis as previously described [13]. Following incubation, cells were washed in PBS (without Ca/Mg) (Gibco) and analysed on a CyAn flow cytometer (Beckman Coulter Life Sciences, Amersham, Buckinghamshire, UK), and the results were analysed using Flow Jo software (Water Biosciences, Wokingham, Berkshire, UK), or cells were fixed onto a coverslip for fluorescence microscopy.

### Neutrophil static adhesion assays

Neutrophils were added to confluent LSECs, which had been stimulated with TNFα 10 μg/ml (Peprotech) for 24 h. The neutrophils (100,000/ml) were left on the LSECs for 5 min in RPMI containing Ca/Mg (Gibco) before removal, and images were captured for analysis. In some experiments LSECs were pre‐conditioned by shear stress (1 kPa for 72 h in an orbital shaker) or transfected with siRNA to knockdown *CLEC14A* (as described above).

### Neutrophil flow adhesion assays

Flow adhesion assays were performed as previously described [14]. Endothelial cells were grown in an Ibidi μ‐slide VI 0.4 channel slide (Thistle Scientific). Once confluent, cells were stimulated with TNF (10 μg/ml; Peprotech) for 24 h (Supplementary materials and methods). If CRT antibodies versus Isotype control were being tested, the antibodies were added to the LSECs at 20 μg/ml along with Fc receptor block 30 min before the flow assay.

### Ischaemia‐reperfusion injury and liver intravital microscopy

Acute liver injury models of ischaemia reperfusion (IR) followed by intravital microscopy were undertaken in 8‐week‐old *Clec14a* KO mice [15]. Hepatic IR injury was induced in mice prior to intravital microscopy, as previously described [16] (Supplementary materials and methods).

### Analysis of CLEC14A expression in publicly available datasets

Enrichment of a neutrophil transcriptomic signature described by Bindea *et al* [17] in human HCC bulk tumour versus paired tumour‐adjacent liver was determined using single‐sample gene set enrichment analysis (ssGSEA). Two publicly available gene expression datasets were analysed: HEPTROMIC (*n* = 228) [18, 19] and The Cancer Genome Atlas (TCGA) (*n* = 361) [20]. Correlation between *CLEC14A* mRNA expression and enrichment of the Bindea neutrophil signature was assessed using the Spearman rank correlation coefficient test in R (version 3.6.1) (https://r-project.org/).

### Spatial transcriptomics

Digital spatial profiling (DSP) was undertaken using NanoString (Bruker Spatial Biology, Bothell, Washington, USA). Cases were selected based on high and low CLEC14A expression from previous chromogenic immunohistochemistry studies. Antigen retrieval was performed using 1× Tris‐EDTA (10 mm Tris, 1 mm EDTA, pH 9.0), and RNA targets were exposed by incubating in Proteinase K solution (Agilent) at 37 °C, followed by washes in 10% neutral buffered formalin (NBF) (CellPath, Newtown, UK), NBF stop buffer (Bruker Spatial Biology), and, finally, PBS. A hybridisation solution was then added to each slide, comprising the RNA panel in Buffer R (GeoMx RNA Slide prep kit, Bruker Spatial Biology) and diethyl pyrocarbonate‐treated H_2_O, and a Grace Bio‐labs HybriSlip (Sigma) was applied. The slides were then incubated overnight at 37 **°**C, washed in saline‐sodium citrate and formamide, and then blocked with Buffer W (GeoMx RNA Slide Prep Kit, Bruker Spatial Biology) for 30 min in a humidity chamber protected from light.

Fluorescent morphology markers Alexa532‐CD3, Alexa594‐CD34, and Alexa647‐PanCK (Thermo Fisher Scientific) were used to visualise the tissue, combined with SYTO13 (Thermo Fisher Scientific) nuclear stain in Buffer W, and added to the tissue before incubating for 1 h in the humidity chamber. Slides were then washed in SCC followed by loading onto the GeoMx DSP.

Regions of interest (ROIs) were selected using the GeoMx DSP analysis suite and labelled as tumour, non‐tumour, or peri‐capsular. Fluorescent markers were added to identify morphology facilitating downstream ROI selection, and profiling reagents were added. These included specific RNA detection reagents, each barcoded with unique photocleavable oligonucleotides. Selected ROIs [21] were confirmed as areas of non‐tumour and tumour with the assistance of a consultant liver histopathologist (OC). ROIs were then exposed to UV light, which led to the decoupling of the oligonucleotides from the profiling reagents. Released oligonucleotides were aspirated into a microtitre plate well, each well representing a single ROI. The oligonucleotide tags were subsequently sequenced, allowing quantification during data processing (Supplementary materials and methods).

### Statistical analyses

All data were tested for normal (Gaussian) distribution using a Shapiro–Wilk normality test. Data presented graphically show mean ± standard error of the mean (SEM) (parametric) or median ± interquartile range (IQR) (non‐parametric) unless otherwise indicated in results. Two independent datasets were compared by Student's unpaired *t*‐test (parametric) or Mann–Whitney test (non‐parametric). Paired data were compared using a Student's paired *t*‐test (parametric) or Wilcoxon matched‐pairs signed‐rank test (non‐parametric). Where appropriate, correlation analysis was performed for parametric or non‐parametric data using a Pearson's or Spearman's correlation test respectively. A *p* value of < 0.05 was considered statistically significant.

## Results

### Upregulation of CLEC14A expression in acute and chronic inflammatory liver conditions

**Figure 1:**
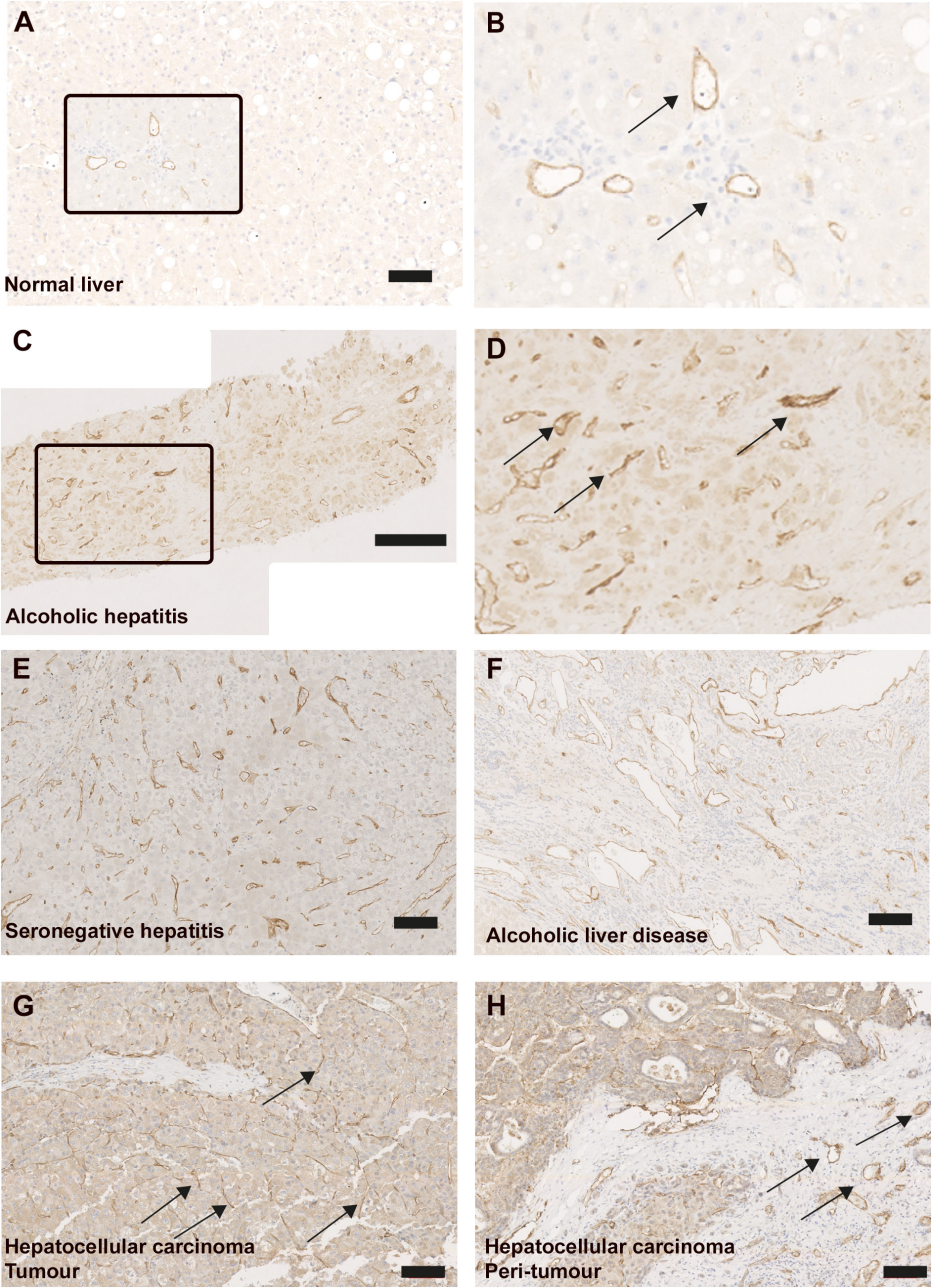
Immunohistochemical analysis of normal and diseased liver. Tissue sections were immunostained for CLEC14A with sheep anti‐human antibody and counterstained with haematoxylin. (A) Image of normal liver (donor liver not used for transplantation). (B) Inset from panel (A) demonstrating CLEC14A expression in a portal tract (black arrows). (C) Biopsy of patient with alcoholic hepatitis. (D) Inset from panel (C) highlighting (black arrows) increased sinusoidal expression of CLEC14A. (E) Sinusoidal expression in a section taken from an explant liver in the setting of acute liver failure secondary to seronegative hepatitis. (F) Scar‐associated vascular expression in a section from an explant liver in the setting of chronic liver disease from alcoholic liver disease. (G, H) Tumour vascular expression of CLEC14A in a section from HCC and peri‐tumoural vessels from a section of HCC (black arrows). Representative images from at least three cases for each condition. Scale bars, 200 μm.

CLEC14A is highly expressed on the vessels of a range of solid organ tumours, including HCC tumours, but its expression on the vasculature of the inflamed liver has not been investigated [9, 11]. We found CLEC14A to be expressed at low levels in the main vessels and sinusoids of the healthy human liver (Figure 1A,B). In liver injury presenting in the acute or chronic setting, we observed marked upregulation of CLEC14A on the liver vasculature. Specifically, high levels of expression were noted in the sinusoidal vasculature of acute inflammatory liver injuries, including alcoholic hepatitis and seronegative hepatitis (Figure 1C–E). CLEC14A expression was also detected in end stage chronic inflammatory liver diseases such as alcoholic liver disease (ALD) (Figure 1F). Consistent with previous studies, CLEC14A was expressed on the tumour vasculature of primary liver cancer both on tumour sinusoidal vessels and peri‐tumoural blood vessels (Figure 1G,H). Thus, while the liver endothelium expresses low levels of CLEC14A in homeostasis, endothelial expression of CLEC14A is substantially upregulated in a range of inflammatory and malignant processes in the diseased human liver.

### CLEC14A expression in tumour vessels correlates with a neutrophil signature and neutrophil infiltration in human hepatocellular carcinoma

**Figure 2:**
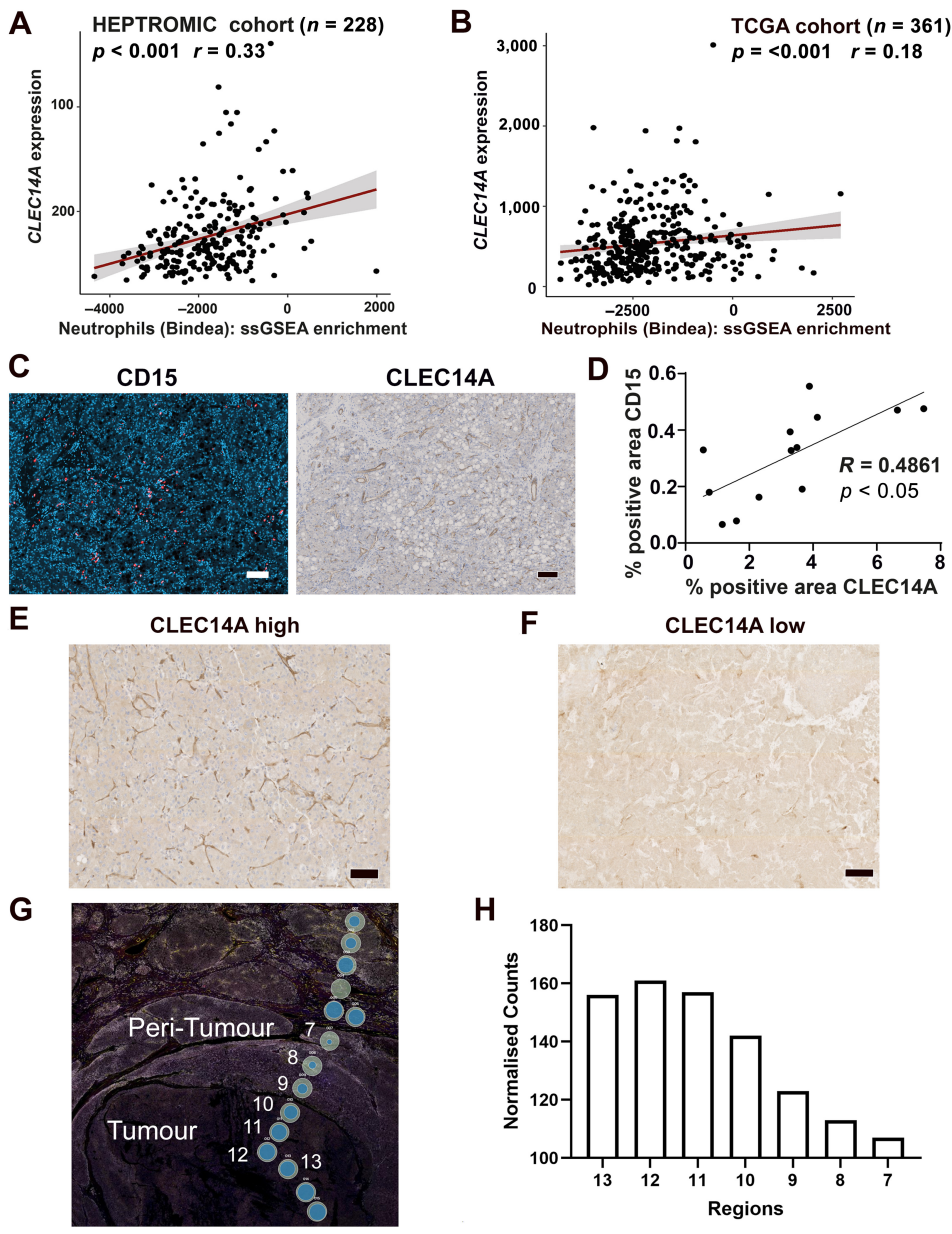
Correlation of CLEC14A with neutrophil gene signature and infiltration in HCC. Correlation analysis of *CLEC14A* expression with an enrichment of a neutrophil signature (Bindea *et al*, [16]), assessed by Spearman rank correlation in (A) HEPTROMIC dataset of HCC bulk sequencing (*n* = 228) and (B) publicly available TCGA HCC cohort (*n* = 361). (C) A cohort (*n* = 15) of surgically resected HCC sections underwent immunostaining for CLEC14A, and matched sequential sections underwent immunofluorescent staining for CD15. (D) Correlation analysis of the percentage of positive area of CLEC14A expression and percentage of positive area of CD15 expression, assessed by Spearman rank correlation coefficient test. (E) Representative image of HCC case with high expression of intratumoural CLEC14A by immunohistochemical staining. (F) Representative image of an HCC case with low expression of intratumoural CLEC14A by immunohistochemical staining. (G) Representative image of ROI selected for spatial transcriptomic analysis for a CLEC14A^high^ case. (H) Corresponding *CLEC14A* transcript expression in selected ROIs taken from panel (G). Scale bars, 100 μm.

Our initial findings led us to perform a more in‐depth exploration of human liver pathologies to specifically explore a potential link between CLEC14A and neutrophils in HCC. Recent phase 3 studies confirmed the efficacy of combining immunotherapy with vascular targeting in HCC, with this strategy exemplified by the combination of atezolizumab and bevacizumab now being the first‐line therapy for advanced unresectable cases [2]. We focused on CLEC14A and its correlation with the tumour immune microenvironment. Initially, we interrogated the HEPTROMIC dataset of HCC bulk sequencing (*n* = 228) for *CLEC14A* and for enrichment of a published neutrophil transcriptomic signature [17]. We found a statistically significant correlation with *CLEC14A* expression in this cohort and validated this correlation in the publicly available TCGA HCC cohort (*n* = 361) (Figure 2A,B).

We then performed quantitative analysis of CLEC14A expression by immunohistochemistry and correlated this with CD15 + neutrophil quantification in matched surgical samples. Our results confirmed a positive correlation between CLEC14A expression and neutrophil infiltration (Figure 2C,D). To support our findings that neutrophil recruitment was a tumour intrinsic process in HCC, we also studied biopsies of HCC to rule out any confounding of operative manipulation or post‐surgical reactive responses. Analysis of freshly collected biopsies demonstrated the presence neutrophil infiltration, confirming their presence in the HCC tissue microenvironment (supplementary material, Figure S1).

Next, we selected a group of CLEC14A^high^ and CLEC14A^low^ tumours for further analysis by ST, with the support of an expert pathologist (OC) (Figure 2E,F). ROIs were selected from the tumour, peri‐tumour, and non‐tumour regions.

Following data normalisation, an initial exploration was performed by generating principle component analysis (PCA) plots for the CLEC14A^high^ and CLEC14A^low^ cohorts (supplementary material, Figure S2A) and PCA analysis of the tumour, peri‐tumour, and non‐tumour selected regions in each cohort (supplementary material, Figure S2B,C). We noted that there appeared to be clearer separation between the non‐tumour regions and tumour regions in the CLEC14A^low^ group compared to CLEC14A^high^ group.

**Figure 3:**
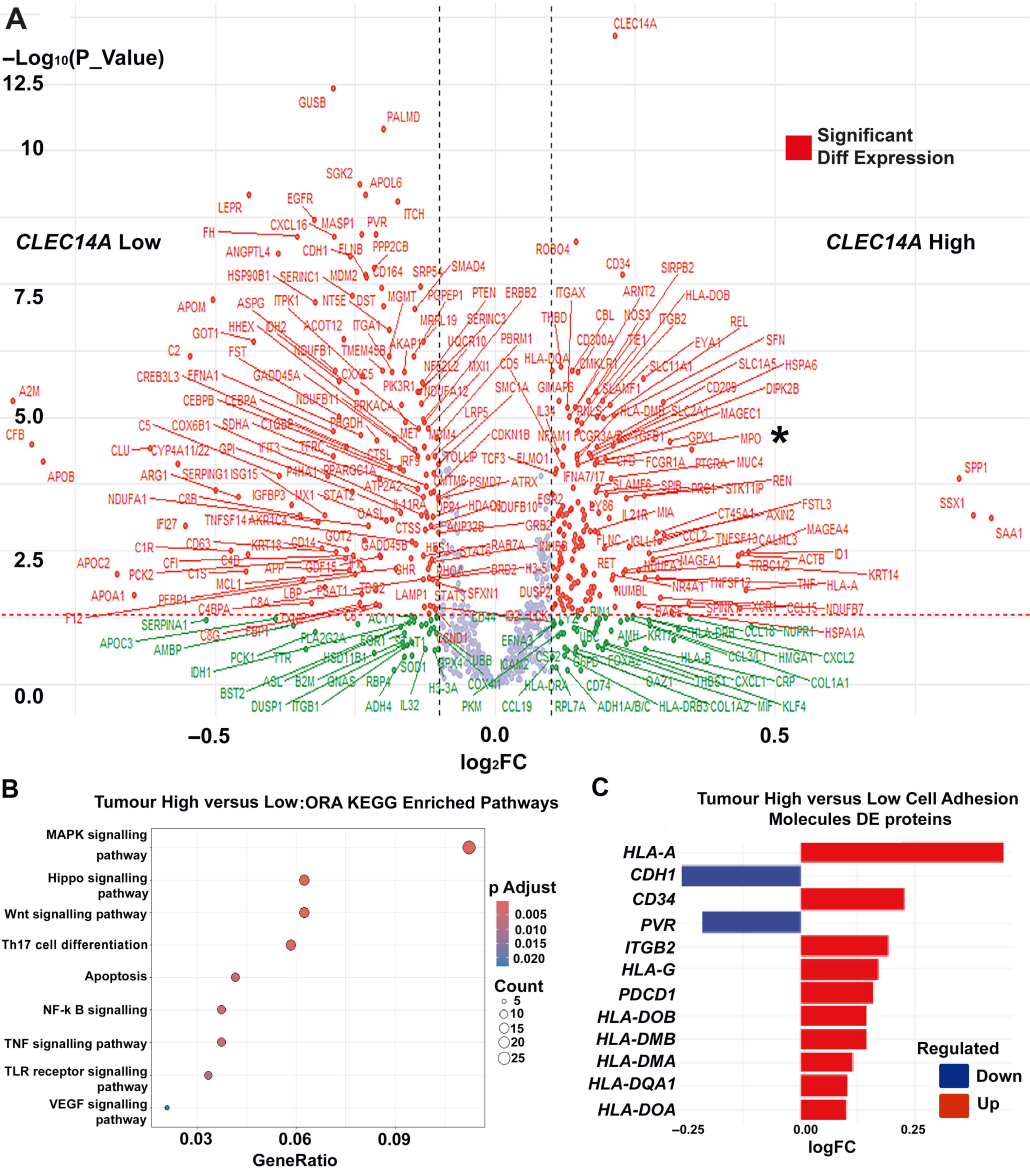
Comparison of gene expression between CLEC14A^high^ and CLEC14A^low^ HCC tumour regions. (A) Volcano plot of differential expression analysis from tumour regions showing differentially expressed genes in *CLEC14A* ^high^ versus *CLEC14A* ^low^ tumours. Asterisk highlights significant upregulation of *MPO*. (B) Pathway enrichment analysis in *CLEC14A* ^high^ tumours reveals upregulation of pathways related to neutrophil activation. (C) Heat map of cell adhesion‐related factors noted to be upregulated (red) and down regulated (blue) in *CLEC14A* ^high^ tumours. Data generated from CLEC14A^high^ tumours (*n* = 4) and CLEC14A^low^ tumours (*n* = 6).

Within tumour regions in CLEC14A^high^ tumours we noted a gradient of *CLEC14A* transcript expression from the centre towards the periphery of the tumour, with the highest expression in the centre (Figure 2G,H). We initially focused on intra‐tumour regions, which were extracted from the ST data, and differential expression analysis was undertaken between CLEC14A^high^ and CLEC14A^low^ tumours (Figure 3A). A total of 165 genes were upregulated in CLEC14A^high^ tumours, and 142 genes were upregulated in CLEC14A^low^ tumours (Figure 3A, red dots). In addition to *CLEC14A* itself, the significantly differentially expressed genes upregulated in CLEC14A^high^ tumours included *ROBO4*, *CD34*, *THBD*, and *CBL*. Notably, the ST data also demonstrated upregulation of neutrophil‐specific gene myeloperoxidase (*MPO*) (Figure 3A, asterisk) in CLEC14A^high^ tumours. To provide an insight into the biological pathways that may distinguish CLEC14A^high^ tumours from CLEC14A^low^ tumours, we undertook Kyoto Encyclopedia of Genes and Genomes (KEGG) pathway enrichment analysis. Figure 3B highlights several upregulated pathways that are related to neutrophil activation in CLEC14A^high^ tumours, including the MAPK pathway and Th17 cell differentiation. Additionally, we analysed our data for cell adhesion molecules, which may contribute to leukocyte recruitment (Figure 3C). We found higher expression of integrin beta2, which is expressed on a range of leukocytes, including neutrophils alongside upregulation of several HLA molecules and CD34 and PD‐1 in CLEC14A^high^ tumours.

**Figure 4:**
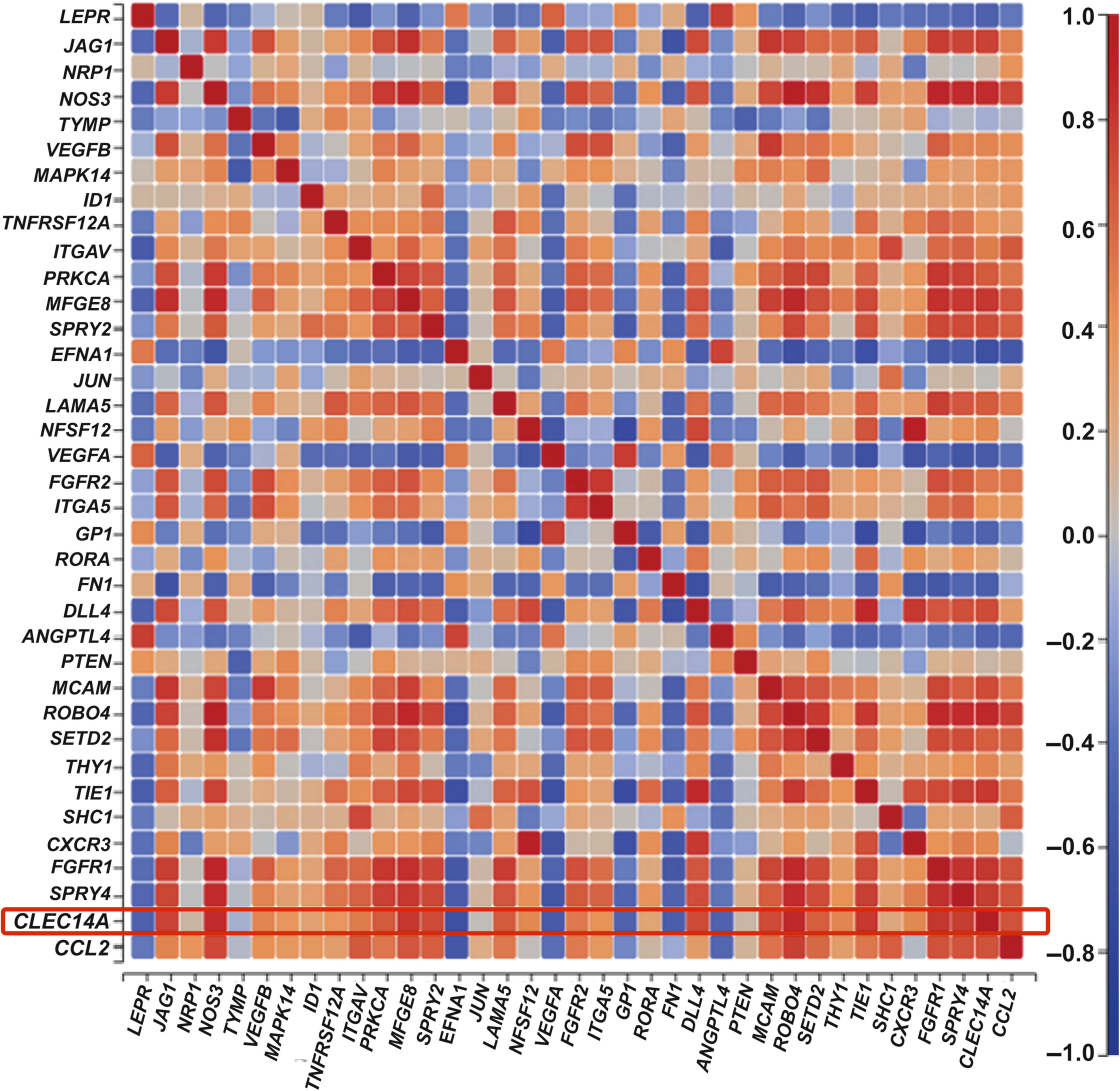
Correlation matrix generated from spatial transcriptomic data for angiogenesis genes in tumour regions from *n* = 10 cases. The red box specifically highlights the correlation of *CLEC14A* with other genes in the panel.

To explore a potential link to angiogenesis in HCC, we studied the angiogenic signature using genes previously grouped to form an angiogenesis panel and included in the Cancer Transcriptome panel provided by Nanostring in tumour and peri‐tumour regions. Within the tumour, a significant positive correlation of *CLEC14A* gene expression was observed with the majority of genes in the angiogenesis panel (24 out of the 36) (supplementary material, Tables S1 and S2; Figure 4).

**Figure 5:**
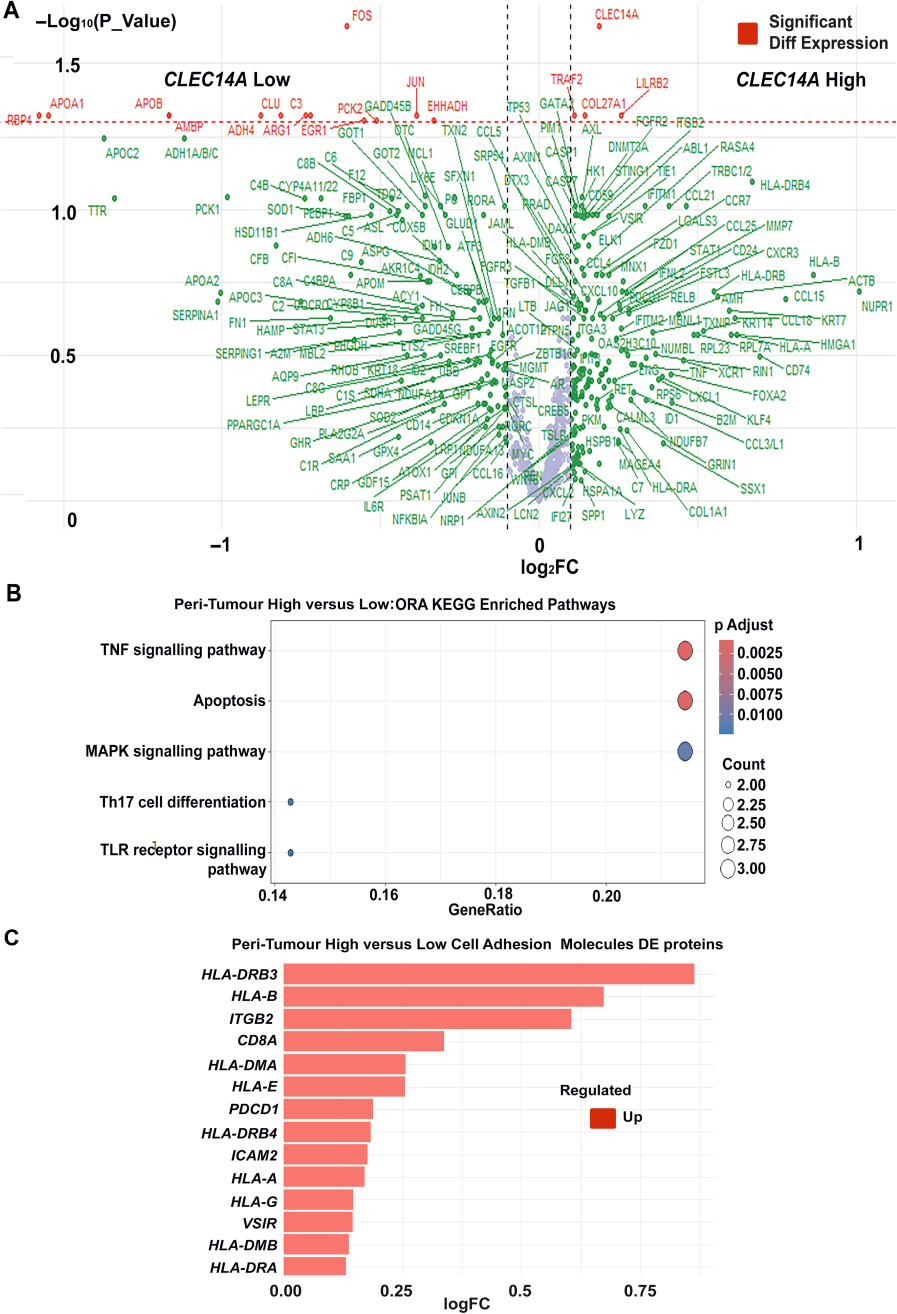
Comparison of gene expression between CLEC14A^high^ and CLEC14A^low^ HCC peri‐tumour regions. (A) Volcano plot of differential expression analysis from peri‐tumour regions comparing *CLEC14A* ^high^ versus *CLEC14A* ^low^ tumours. (B) Pathway enrichment analysis of differentially expressed genes in peri‐tumour region of *CLEC14A* ^high^ tumours reveals upregulation of TNF and MAPK pathways. (C) Analysis of cell adhesion‐related factors demonstrate upregulation in the peri‐tumour regions of CLEC14A^high^ tumours.

ST analysis also enabled analysis of differential expression in the peri‐tumour regions of CLEC14A^high^ tumours to CLEC14A^low^ tumours. Here, we found significantly fewer genes differentially expressed compared to the tumour regions (Figure 5A). Despite this, a selected group of inflammatory pathways were upregulated, as identified by KEGG pathway enrichment, including the TNF signalling pathway and the MAPK signalling pathway (Figure 5B). We also found that the cell adhesion molecules upregulated were not only *ITGB2* and *PDCD1* as detected in the CLEC14A^high^ tumour regions, but also *CD8* and *ICAM2* (Figure 5C).

### CLEC14A is expressed by primary human LSECs *in vitro* and mediates their angiogenic properties

To complement our pathological findings, we aimed to explore the functional role of CLEC14A in liver endothelium. We isolated primary human LSECs as previously described [22]. CLEC14A expression on endothelial cells is regulated by shear stress, and the low shear stress found in tumour vasculature may contribute to the upregulation of CLEC14A [9]. We confirmed these previous findings in primary human liver endothelium with real‐time PCR in static‐ and shear‐exposed conditions and also found that hypoxia, a characteristic of the chronically inflamed liver as well as the TME, was associated with upregulated *CLEC14A* expression (supplementary material, Figure S3A,C). Interestingly, transcriptional regulation of *MMRN2* showed the opposite trend, with upregulation at high shear stress and downregulation with hypoxia (supplementary material, Figure S3B,D).

We next studied the protein expression of CLEC14A in LSECs isolated from healthy and cirrhotic livers. CLEC14A was detectable by western blotting in LSEC lysates taken from both healthy and cirrhotic livers (supplementary material, Figure S4A). Notably, in non‐reducing conditions, CLEC14A was detected in two bands. Total quantification of these bands demonstrated higher expression of CLEC14A in diseased liver endothelium compared to healthy livers, in keeping with our immunohistochemical analysis. The two bands detected were at 100 and 250 kDa. Previous studies have shown that the mature glycosylated form is detectable at 100 kDa in human umbilical vein endothelial cells (HUVECs) [9]. We hypothesised that the higher band was detected due to CLEC14A binding to its known ligand multimerin‐2 (MMRN2), and this interaction was maintained in non‐reduced conditions. To confirm this, the blot was reprobed for MMRN2, which clearly demonstrated detection of MMRN2 at the 250 kDa mark (supplementary material, Figure S4B). This result suggests that CLEC14A in human liver endothelium can be detected in two separate states, either as bound or unbound to MMRN2. We repeated the western blotting under reducing conditions in which the interaction between CLEC14A and MMRN2 was disrupted, such that CLEC14A was detected only at a molecular weight of 100 kDa (supplementary material, Figure S4C).

To explore the functional role of CLEC14A in LSECs, we used siRNA knockdown and confirmed knockdown with two duplexes (supplementary material, Figure S4D). In HUVECs, CLEC14A has been shown to mediate angiogenesis [15]. We therefore performed angiogenesis assays in LSECs. Knockdown of *CLEC14A* led to a trend in reduced tube formation in Matrigel assays, which was significant at 6 h but not 12 h (supplementary material, Figure S4E,F). These results led us to explore additional functional roles of CLEC14A in LSECs.

### CLEC14A mediates neutrophil adhesion to human LSECs under conditions of low shear stress independent of the CLEC14A/MMRN2 interaction

**Figure 6:**
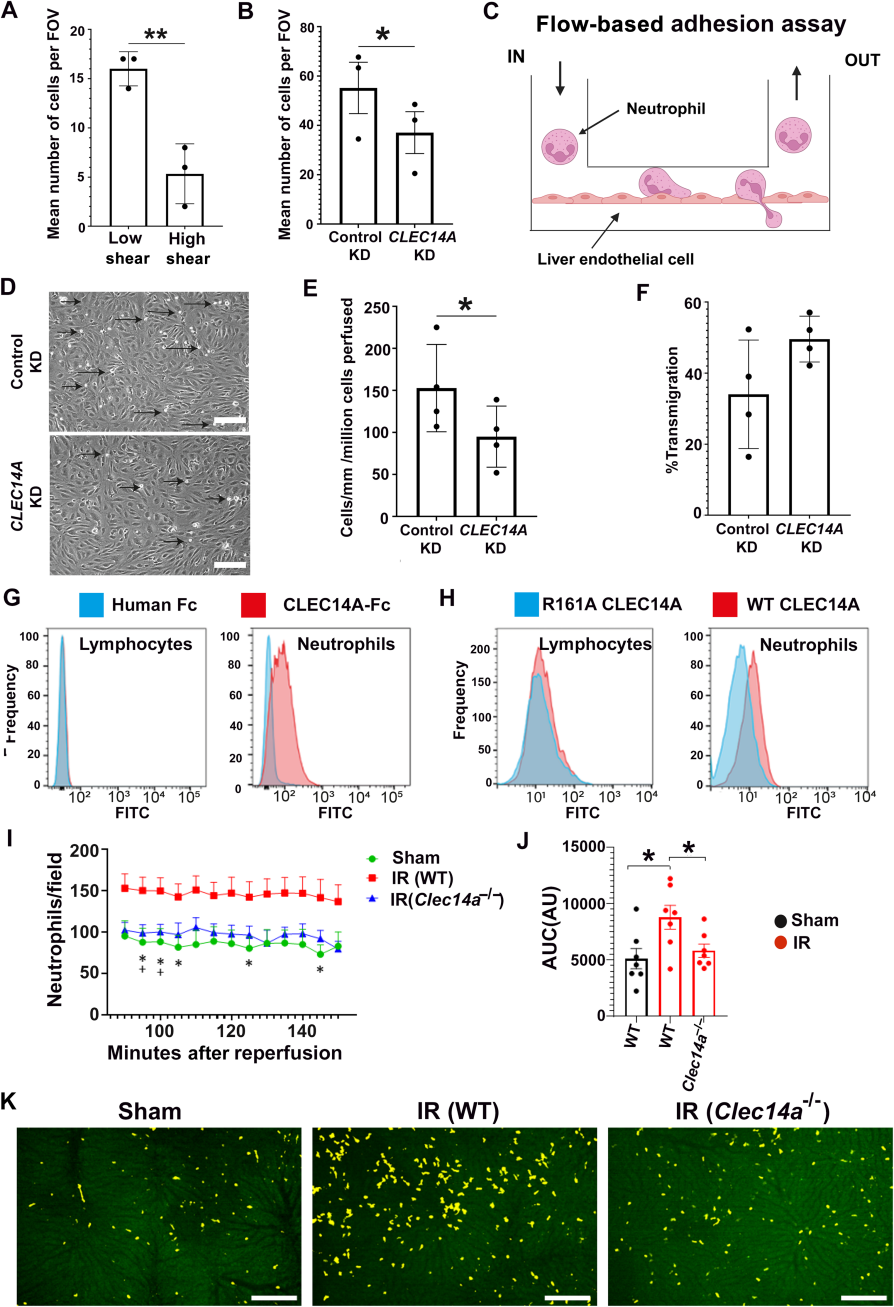
The role of CLEC14A in neutrophil recruitment across liver sinusoidal endothelial cells. (A) Human LSECs were cultured under standard static conditions or exposed to shear stress for 72 h, followed by incubation with peripheral blood neutrophils to allow adherence, and then unadhered neutrophils were removed, at least 10 fields of view were selected avoiding the outer edges and centre of the plates. The number of adhered neutrophils was counted each field of view, *n* = 3. (B) LSECs were cultured under static conditions either with mock/scrambled siRNA knockdown or *CLEC14A* knockdown and adhesion assays repeated with peripheral blood neutrophils (*n* = 3). (C) Flow adhesion assays were performed with neutrophils perfused over a monolayer of LSECs at a shear stress of 0.05 Pa to recapitulate the liver sinusoids. Created in BioRender. Shetty, S. (2026) https://BioRender.com/5pm8cvp. (D) Representative images of neutrophils adherent to mock siRNA (upper panel) or *CLEC14A* siRNA knockdown (lower panel) taken by phase contrast microscopy. Scale bar, 50 μm. (E, F) Adhered and transmigrated (as a percentage of total adherent) neutrophils were quantified in 10 visual fields per lane (*n* = 4). For panels (A–F), the results are shown as mean ± SEM, and significance from a paired *t*‐test is displayed *p < 0.05 and **p < 0.01. (G) Flow cytometry of peripheral blood lymphocytes (left panel) and neutrophils (right panel) incubated with fluorescently labelled Human‐Fc (blue) or Recombinant CLEC14A‐Fc (red). (H) Flow cytometry of peripheral blood lymphocytes (left panel) and neutrophils (right panel) incubated with fluorescently labelled heparin binding mutant CLEC14A (R161A) (blue) or WT recombinant CLEC14A (red). (I, J) *Clec14a* KO and WT mice underwent ischaemic reperfusion (IR) injury with quantification of neutrophil recruitment following reperfusion injury to the liver sinusoids,(I) cells per field of view following IR in WT and *Clec14a*‐KO animals, and Sham treated WT mice and (J) area under the curve analysis Sham, IR in WT and *Clec14a*‐KO animals. (K) Representative images of the liver sinusoids following reperfusion where neutrophils (pre‐labelled with anti GR‐1 antibody, yellow) can be visualised in the sinusoidal vascular bed (green is autofluorescence). Scale bar, 200 μm. All groups *n* = 7; panel (I): mean ± SEM, two‐way ANOVA followed by Dunnett's post‐test against WT IR, * *p* < 0.05 WT IR versus sham, + *p* < 0.05 WT IR versus IR (C*lec14a* ^−/−^). Panel (J): mean ± SEM, one‐way ANOVA followed by Holm–Sidak's multiple‐comparisons test, *p < 0.05.

The potential of vascular targeting as a novel approach to immunomodulation has been highlighted elsewhere [2, 3]. We initially undertook neutrophil adhesion assays with LSEC monolayers pre‐conditioned by high shear stress and compared them to LSECs cultured in static conditions. This confirmed that neutrophil adhesion was significantly inhibited by shear stress exposure, which is known to downregulate CLEC14A (Figure 6A). To determine a specific role for CLEC14A in the process of neutrophil adhesion, static adhesion assays were repeated in the setting of *CLEC14A* knockdown. We confirmed a significant reduction in neutrophil adhesion to LSECs when CLEC14A expression was experimentally downregulated (Figure 6B). To recapitulate organ‐specific recruitment in the liver sinusoids, we undertook flow‐based adhesion assays, which permit the perfusion of neutrophils and visualisation of each step of the adhesion cascade with phase contrast microscopy [14] (Figure 6C). With *CLEC14A* knockdown, we observed a significant reduction in the firm adhesion step of neutrophil recruitment to LSECs (Figure 6D,E), but not in the transmigration step (Figure 6F). We next examined the potential role of the known ligand for CLEC14A, MMRN2, and incorporated two monoclonal antibodies (CRT 4 and 5) that have been shown to block the interaction of MMRN2 with CLEC14A [15]. Compared to isotype‐matched controls, no differences in neutrophil recruitment were observed when the CLEC14A/MMRN2 interaction was blocked (supplementary material, Figure S5), suggesting that CLEC14A regulation of neutrophil recruitment was independent of its interaction with MMRN2.

To further explore whether CLEC14A could bind to neutrophils directly, we incubated fluorescently labelled protein with lymphocytes and neutrophils. Flow cytometric analysis demonstrated binding of recombinant CLEC14A‐Fc to the surface of isolated peripheral blood neutrophils compared to human‐Fc, but not to peripheral blood lymphocytes (Figure 6G). Previous proteomic‐based screens of the endothelial heparan sulphate interactome confirm that CLEC14A also binds heparin [13]. We tested whether this binding site may regulate neutrophil binding to CLEC14A by repeating experiments with mutant CLEC14A, where residue 161 (heparin binding site) was substituted (R161A) to abrogate heparin binding [13]. Binding of CLEC14A to lymphocytes was similar between mutant and WT sequences; however, the R161A substitution led to a dramatic reduction in the binding of CLEC14A to neutrophils (Figure 6H).

### *Clec14a* KO reduces neutrophil recruitment in an acute liver injury model

To determine a role for CLEC14A in neutrophil recruitment within a multicellular environment, we undertook an *in vivo* model of IR injury of the liver in *Clec14a‐*deficient mice. *Clec14a*‐deficient mice were previously described and studied by our group [15]. We chose the IR model as a well‐established model for studying neutrophil recruitment to the liver [23]. This model is characterised by significant recruitment of neutrophils via the liver sinusoids during the reperfusion phase. We undertook these models in the setting of *Clec14a* KO and compared them to age‐ and sex‐matched WT controls. As described previously, these experiments demonstrated robust recruitment of neutrophils to the hepatic sinusoids in WT mice during reperfusion injury compared to sham injury. In comparison, *Clec14a* KO mice were found to have a significantly attenuated recruitment of neutrophils within the liver sinusoids (Figure 6I–K). These *in vivo* results therefore provide complementary evidence in a multicellular environment to support our earlier human *in vitro* studies.

## Discussion

In recent years, the HCC treatment landscape has changed with the approval of immune checkpoint therapy in combination with VEGF blockade [2]. This has also led to gathering interest in targeting endothelial cells as a partner with immunotherapy to help shape the infiltration and activation of leukocyte subsets within the TME [3].

We found that two publicly available datasets confirmed that *CLEC14A* ^high^ tumours were characterised by a stronger neutrophil signature compared to tumours with low expression of *CLEC14A*. ST provided more in‐depth analysis of tumours, initially confirming the highest transcriptional *CLEC14A* signature at the centre of the tumour. This may reflect findings from previous studies demonstrating reduced perfusion in the centre of tumours and increased blood stasis [24, 25]. Subsequent gene expression analysis did identify pathway differences between CLEC14A^high^ tumours compared to CLEC14A^low^ tumours with overall a more pro‐inflammatory signature in CLEC14A^high^ tumours. Similar findings were also noted in the peri‐tumoural regions of CLEC14A^high^ samples, although gene expression differences were minimal compared to the intra‐ tumour regions. While cell adhesion factors were not among the most significantly expressed genes in CLEC14A^high^ tumours, specific leukocyte recruitment molecules were noted to be upregulated, including *ITGB2* and *ICAM2*, both of which have been implicated in neutrophil/endothelial interactions [26, 27].

Further analysis confirmed that CLEC14A^high^ tumoural expression correlated with several angiogenic genes that are also known to be upregulated by low shear stress, including *ROBO4*, *DLL4*, and *TIE1* (supplementary material, Table S1) [28, 29, 30]. Interestingly, while there was a positive correlation with *VEGFB* and VEGF signalling, there was a negative correlation with *VEGFA* (Figure 4; supplementary material, Table S1). Previous studies demonstrated that CLEC14A deficiency increased VEGFA‐dependent endothelial cell sprouting and vessel density and loss of CLEC14A enhanced VEGFR‐2 expression, so an inverse relationship may be plausible [31]. These results will support further studies exploring whether CLEC14A may be a biomarker or an alternative therapeutic target for patients who do not respond to VEGF inhibition due to low VEGF‐A tumoural expression.

To further explore a direct functional role for CLEC14A in neutrophil infiltration, we undertook specific neutrophil recruitment studies. Neutrophil recruitment follows a complex cascade of receptor–ligand interactions that leads to rolling and firm adhesion, followed by transendothelial migration [6]. Previous work showed that the liver is a unique environment for leukocyte recruitment [32]. We provide new insights into this process by demonstrating that the shear‐regulated receptor CLEC14A contributes to neutrophil–endothelial interactions in the liver's specialised low‐flow vascular beds. CLEC14A was upregulated on endothelial populations in both acute and chronic liver inflammation, as well as HCC tumour vasculature. We then confirmed a functional role for CLEC14A in neutrophil recruitment across endothelial cells in our flow‐based adhesion assay using siRNA knockdown of primary LSECs, as well as in an *in vivo* model of liver injury in a *Clec14a* KO. Here, we showed that CLEC14A contributed to the firm adhesion step of the neutrophil adhesion cascade.

Thus, CLEC14A may represent a new therapeutic target for neutrophil recruitment, specifically in the context of low shear stress. Mechanistically, building on previous studies that CLEC14A was also a heparin binding protein, we showed that a mutant form of CLEC14A with an altered heparin binding domain impaired neutrophil binding, suggesting that this region regulates neutrophil interactions. Further work is required to confirm a ligand binding partner expressed on neutrophils and to determine whether CLEC14A directly binds to neutrophils *in vivo* during recruitment to the liver.

A limitation of our study is the focus on neutrophil recruitment. We did not perform functional studies on how CLEC14A may regulate neutrophil phenotype, especially given the growing interest in neutrophil heterogeneity and plasticity. Therefore, this should be the focus of future CLEC14A studies.

## Author contributions statement

JMO, DAP, DK, GN, KS and KY performed data acquisition, data analysis and interpretation. AO, CEW, KH, JF and JLM performed data acquisition. OC performed histological evaluation. HLR, DAM and JML contributed to study concept, funding acquisition and critical revision of the manuscript. YTM, TH and CJW undertook data acquisition and supervision and critical revision of the manuscript. PWH, NK, FS, AJN, VH, RB and SS contributed to study concept, supervised the experiments and helped write the manuscript. JMO and SS accessed and verified the underlying data. All authors read and approved the final version of the manuscript.

## Supporting information


Supplementary materials and methods
**Figure S1.** Immunohistochemical analysis of neutrophil markers CD66b (top panel) and MPO (bottom panel) in tumour biopsies taken from patients with hepatocellular carcinoma
**Figure S2.** Spatial transcriptomic analysis in CLEC14A^high^ and CLEC14A^low^ HCC cases
**Figure S3.** Isolation of primary LSECs from whole liver tissue and qPCR analysis under normoxic and hypoxic conditions
**Figure S4.** Western blotting of CLEC14A in primary human LSECs
**Figure S5.** Flow adhesion assays performed with neutrophils perfused over primary LSECs
**Table S1.** Correlation values for genes associated with angiogenesis in tumour areas
**Table S2.** Correlation values for genes associated with angiogenesis in peri‐tumour areas

## Data Availability

RNA sequencing data are available through the Gene Expression Omnibus: GSE253962 https://www.ncbi.nlm.nih.gov/geo/query/acc.cgi?acc=GSE253962
