# Supplementary material for: CLEC14A correlates with neutrophil infiltration in hepatocellular carcinoma and mediates neutrophil recruitment across liver endothelial cells
Source: J Pathol. 2026 Jun 1;269(4-5):478–92. doi: 10.1002/path.70077 (PMC13341266; doi:10.1002/path.70077)
Supplement: Supplementary file 1 — Supplementary materials and methods Figure S1. Immunohistochemical analysis of neutrophil markers CD66b (top panel) and MPO (bottom panel) in tumour biopsies taken from patients with hepatocellular carcinoma Figure S2. Spatial transcriptomic analysis in CLEC14Ahigh and CLEC14Alow HCC cases Figure S3. Isolation of primary LSECs from whole liver tissue and qPCR analysis under normoxic and hypoxic conditions Figure S4. Western blotting of CLEC14A in primary human LSECs Figure S5. Flow adhesion assays performed with neutrophils perfused over primary LSECs Table S1. Correlation values for genes associated with angiogenesis in tumour areas Table S2. Correlation values for genes associated with angiogenesis in peri‐tumour areas [file PATH-269-478-s001.docx]

**CLEC14A correlates with neutrophil infiltration in hepatocellular carcinoma and mediates neutrophil recruitment across liver endothelial cells**

JM O'Rourke , *et al. J Pathol* <https://doi.org/10.1002/path.70077>

**Supplementary materials and methods**

**Supplementary Figures S1–S5**

**Supplementary Tables S1 and S2**

**Supplementary materials and methods**

**Flow assay analysis**

Neutrophils were perfused over the LSECs at a concentration of 500,000 neutrophils/ml at 0.05 Pa for 5 min at 37 °C. Neutrophils were kept in RPMI free from Ca/Mg (Gibco, Paisley, Renfrewshire, UK) until being added to medium containing Ca/Mg at the start of each lane run. After 5 min of flow, the LSECs were washed with flowing medium for 3 min. Adherent neutrophils were captured using a phase-contrast Olympus IX50 inverted microscope (Olympus, Southend-on-Sea, Essex, UK). Adherent neutrophils appear phase bright, and those that had transmigrated appear phase dark. Adhesion was converted to cells per square millimetre and corrected for the number of neutrophils perfused (i.e., adherent cells/mm^2^/10^6^ perfused).

**Hepatic ischaemic-reperfusion injury**

In brief, the arterial and venous supplies to the left hepatic lobe were clamped for 90 min to induce partial hepatic ischaemia in either CLEC14A KO mice or age/sex-matched WT controls. Five minutes prior to reperfusion, mice received 20 µl PE-conjugated anti-Gr-1 (diluted in 80 µl PBS; Clone RB6-8C5; eBioscience, Paisley, UK) via a carotid cannula to label neutrophils. To initiate reperfusion, the clamp was removed and the liver was allowed to reperfuse for 90 min prior to transfer to an Olympus IX81 Nipkow spinning-disk confocal intravital microscope (Olympus). Control animals underwent sham surgery. The liver was imaged once every 10 s for 1 h by exposure to excitation light (200 ms), starting at 90 min after reperfusion. Neutrophils were analysed by automated object tracking (ImageJ version 1.53t, NIH; <https://imagej.net/ij/index.html>) and analysis performed every 5 min.

**Spatial transcriptomics data processing and analysis**

***Data processing***

Following library preparation and sequencing, BCL files were demultiplexed into FASTQ files using bcl2fastq (version 2.2, Illumina, Cambridge, UK). FASTQ files were subsequently processed to compatible DCC files via the GeoMxNGSPipeline (version 2.2, Bruker Spatial Biology, Bothell, WA, USA). DCC files containing the counts for the experiment were uploaded to the GeoMx Analysis Suite, and a study was created with a file containing annotations relevant to the ROIs, for example, denoting whether a particular ROI was tumour, peri-tumour, or non-tumour. Selected genes were chosen from the Cancer Transcriptomic Atlas panel and contained 1,800 targets. There were five different probes per gene in the RNA panel applied to the tissue at hybridisation. In total, 12 tumours (six CLEC14^high^ samples and six CLEC14A^low^ samples) and adjacent non-tumour tissues were processed for the experiment. For two of the CLEC14A^high^ tumours, the sequencing data did not pass quality control (due to the low number of counts per spatial transcriptomic spot). The remaining 10 cases were carried forward for analysis.

**Spatial transcriptomic data analysis**

PCA of the spatial transcriptomic data from tumour, peri-tumour, and non-tumour regions was performed using the scikit-learn library, version 1.3.0 in Python.

After applying a log transformation to the data, quantile normalization across arrays was performed using the *normalizeBetweenArrays()* function of linear models for the microarray data (LIMMA) package in R-Bioconductor (DOI: [10.1214/16-AOAS920](https://projecteuclid.org/journals/annals-of-applied-statistics/volume-10/issue-2/Robust-hyperparameter-estimation-protects-against-hypervariable-genes-and-improves-power/10.1214/16-AOAS920.full)). Subsequently, samples from the tumour region and samples from the peri-tumour region were analysed separately. LIMMA was used to find differentially expressed genes between CLEC14A^high^ samples and CLEC14A^low^ samples in the same region. *P* values were adjusted using the Benjamini–Hochberg (BH) method, and only genes with |logFC|>0.1 and BH-adjusted *P* values < 0.05 were considered significantly differentially expressed and were used for the downstream KEGG pathway enrichment analysis.

KEGG pathway enrichment analysis of the significantly differentially expressed genes was performed using the *enrichKEGG()* function of the ‘clusterProfiler’ R package (DOI: [10.1007/978-1-0716-0849-4_9](https://link.springer.com/protocol/10.1007/978-1-0716-0849-4_9)).

GSEA was conducted using the *gseKEGG()* function. *P* values were adjusted using the BH method, with a significance threshold set at <0.05.

**
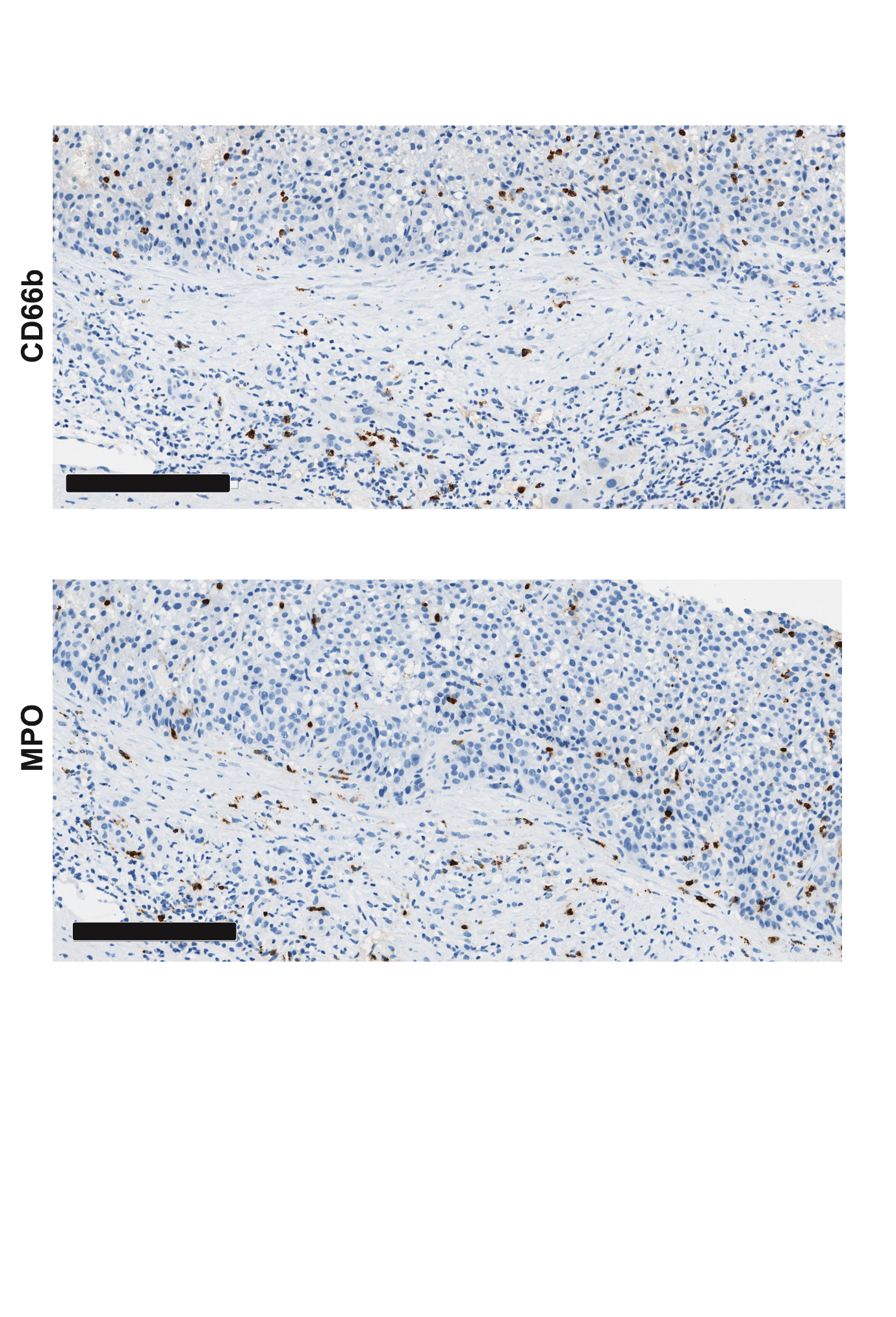
**

**Figure S1. Immunohistochemical analysis of neutrophil markers CD66b (top panel) and myeloperoxidase (MPO) (bottom panel) in tumour biopsies taken from patients with hepatocellular carcinoma.** Scale bar, 200 µm

**
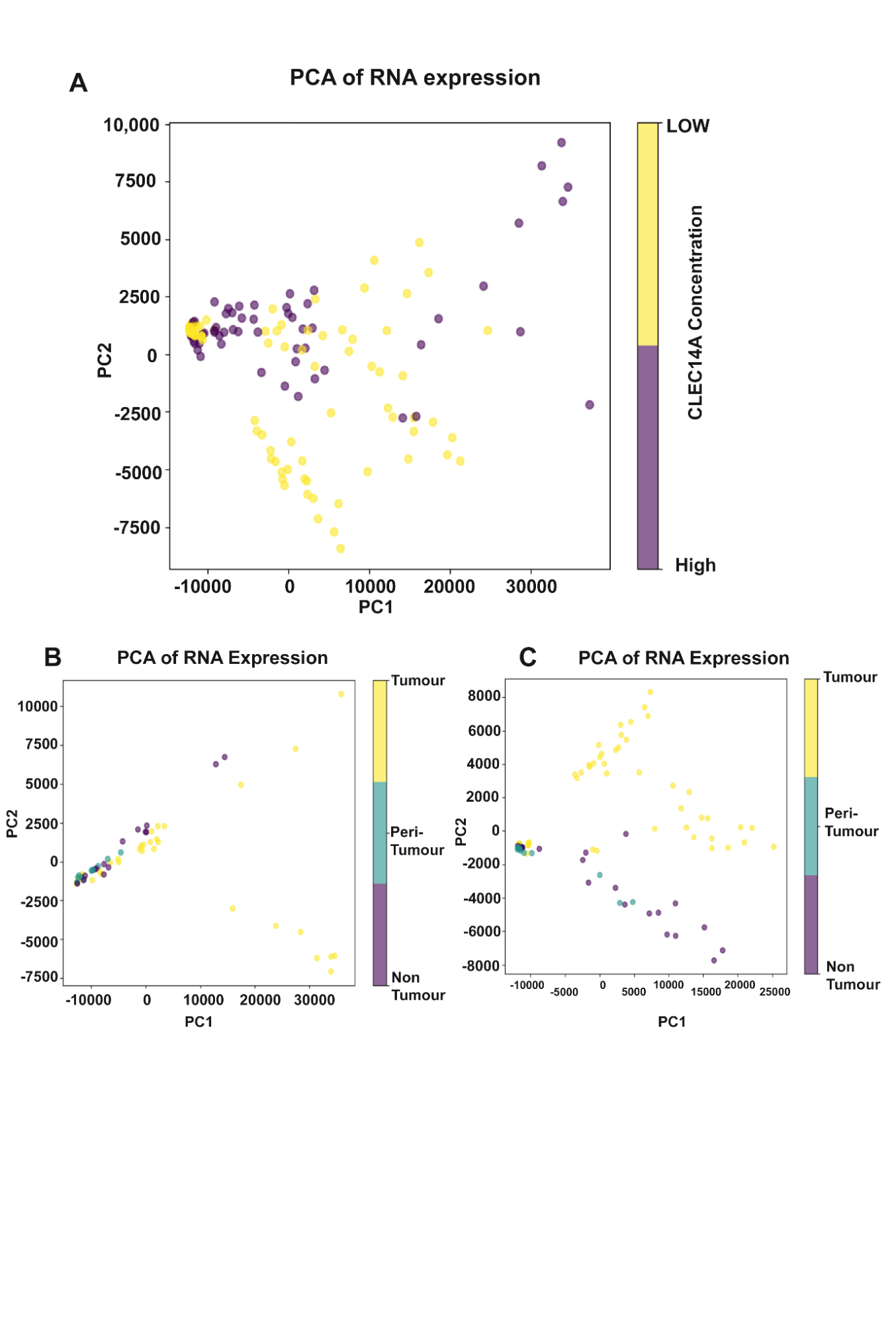
**

**Figure S2. Spatial transcriptomic analysis in CLEC14A^high^ and CLEC14A^low^ HCC cases.** PCA plots are shown for regions selected in each case following data normalisation. (A) Comparison of tumours classified as CLEC14A high and low. (B) Comparison of tumour, peri-tumour, and non-tumour regions in CLEC14A^high^ cases. (C) Comparison of tumour, peri-tumour, and non-tumour regions in CLEC14A^low^ cases.

**
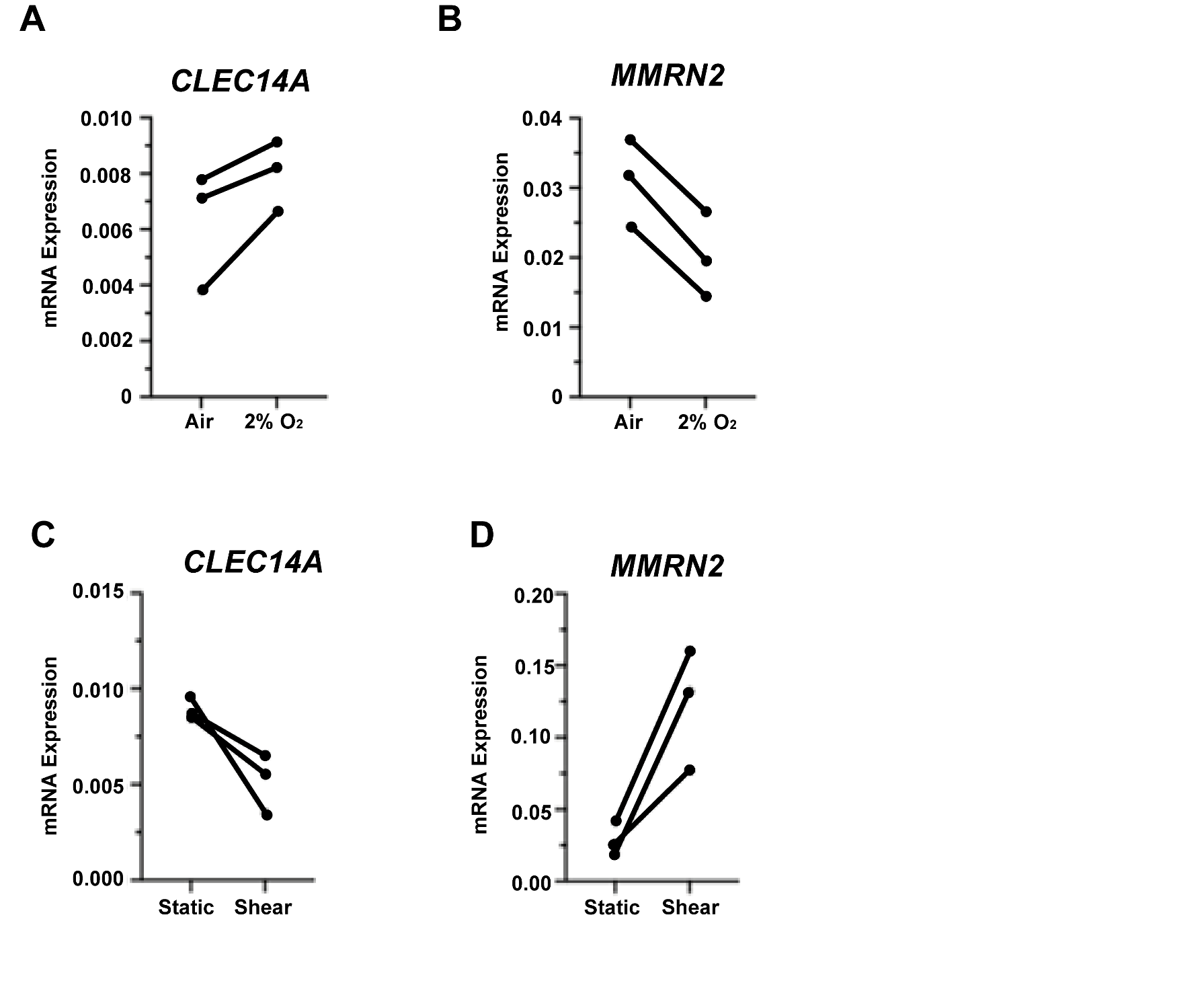
**

**Figure S3**. **Isolation of primary LSECs from whole liver tissue and qPCR analysis under normoxic and hypoxic conditions.** (A and B) qPCR of LSECs exposed to normoxic and hypoxic conditions for *CLEC14A* and *MMRN2*. (C and D) qPCR of LSECs exposed to static and high shear stress conditions for 24 h for *CLEC14A* and *MMRN2*. Relative expression to housekeeping gene ACTB. *n*= 3 for each experiment.

**
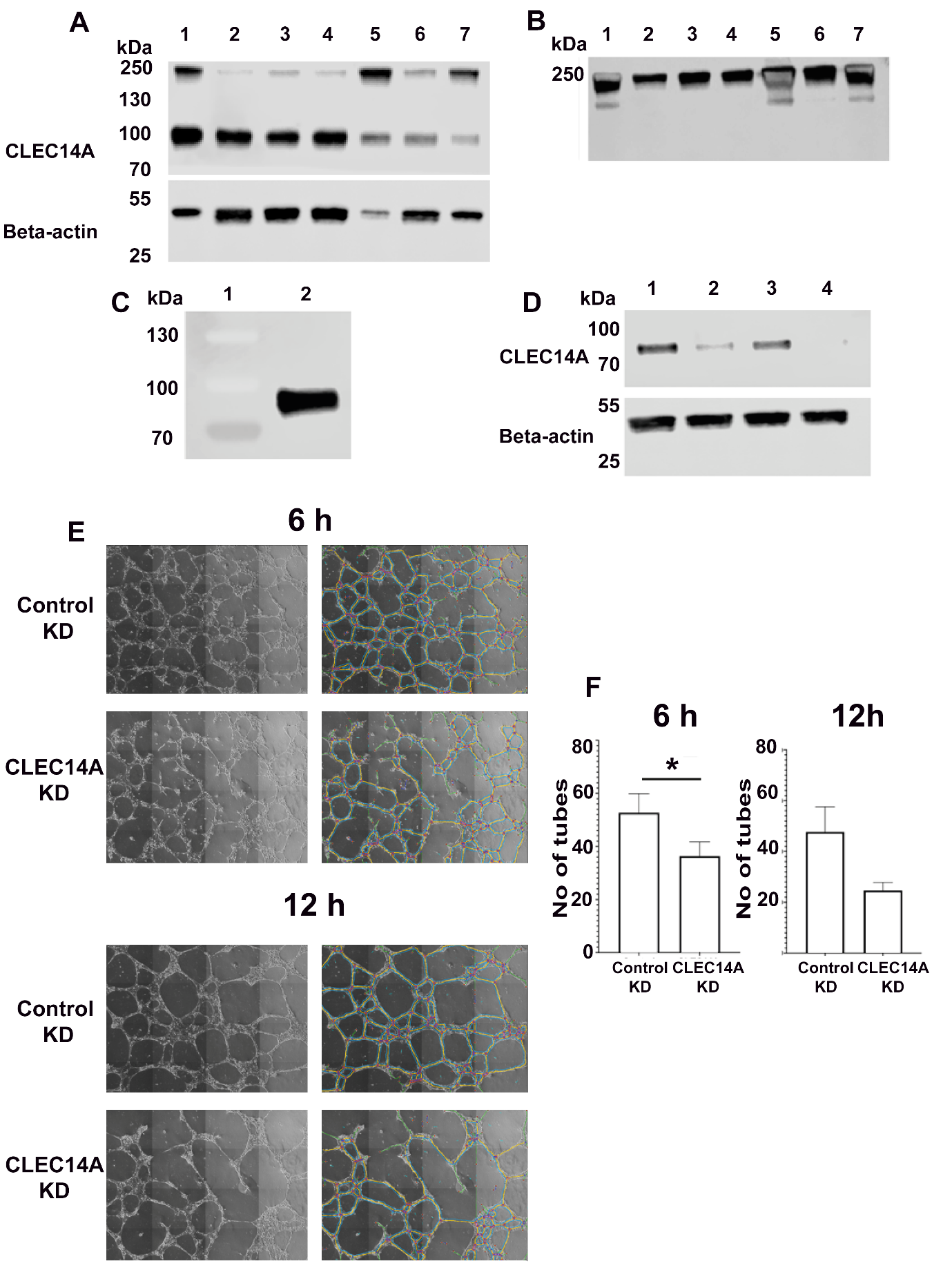
**

**Figure S4. Western blotting of CLEC14A in primary human liver sinusoidal endothelial cells (LSECs).** (A) LSEC samples isolated from chronically diseased liver tissue (established cirrhosis, lanes 1–5) and from normal liver (lanes 6 and 7) for CLEC14A in non-reducing conditions. (B) Reprobing of western blotting from panel (A) with a primary antibody for MMRN2 (lanes 1–7). (C) Western blotting for CLEC14A in LSECs in reducing conditions demonstrating a single band at 100 kDa (lane 2). (D) Western blotting showing that two independent siRNA duplexes (scrambled control siRNA, lanes 1 and 3; and *CLEC14A* knockdown, lanes 2 and 4) efficiently knock down CLEC14A in human LSEC. Beta actin served as the loading control. (E) Representative montage images of Matrigel tube formation assay. Top four panels: 6-h time point (scrambled siRNA, upper two panels; *CLEC14A* knockdown, lower two panels). Bottom four panels: 12-h time point (scrambled siRNA, upper panels; *CLEC14A* knockdown, lower two panels). For both time points, phase contrast images are shown on the left and post-analysis images on the right indicating tubes and nodes. (F) Quantification of tube formation at 6- and 12-h time points for human LSECs transfected with scrambled (control) or *CLEC14A* siRNA duplexes from three independent experiments.

**
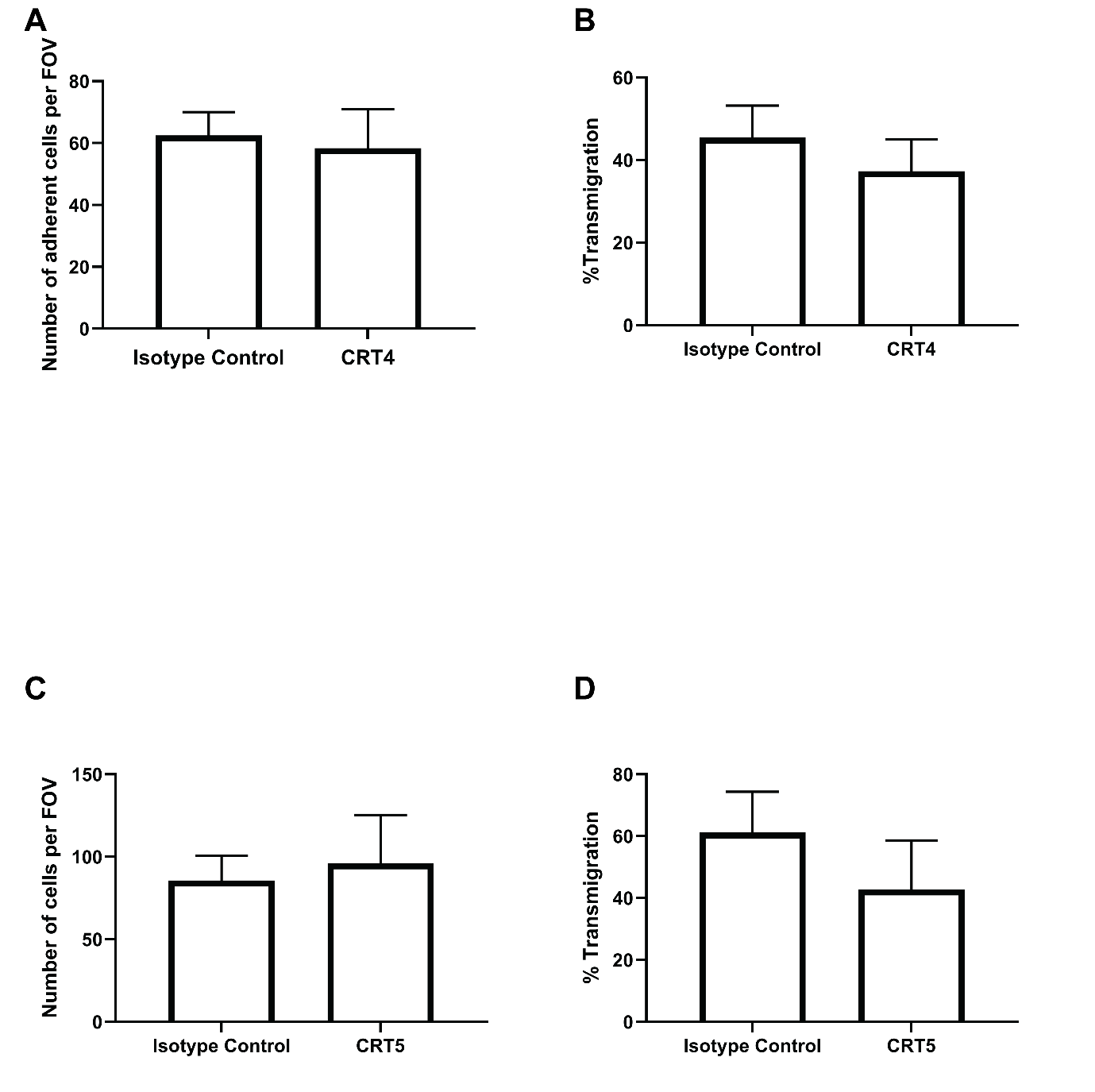
**

**
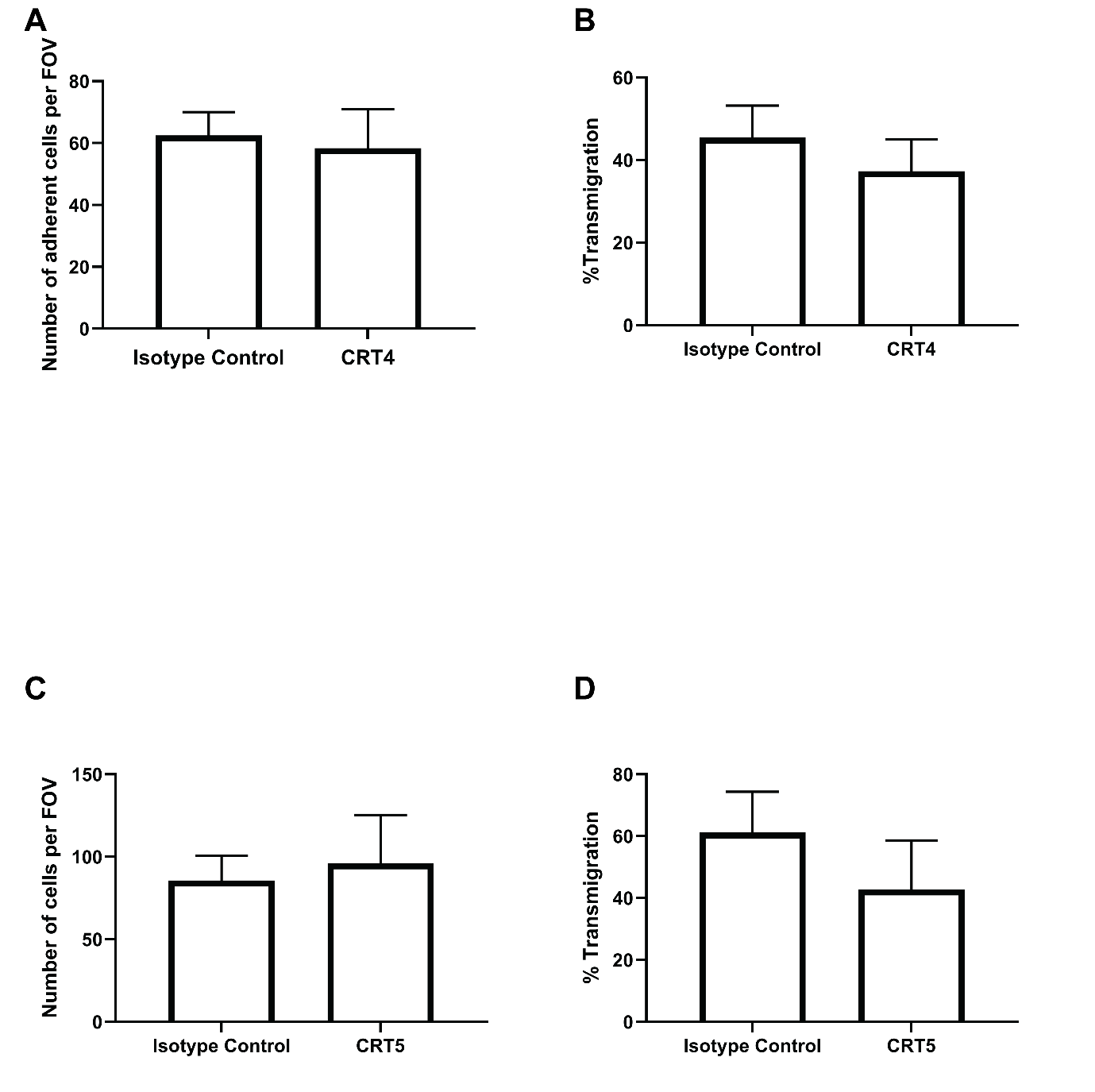
**

**Figure S5.** **Flow adhesion assays with neutrophils perfused over primary liver sinusoidal endothelial cells (LSECs).** Endothelial monolayers were pre-treated with monoclonal antibodies previously shown to target the binding site of MMRN2 on CLEC14A: (A and B) CRT-4 and (C and D) CRT-5. Neutrophil recruitment was compared to LSECs treated with isotype-matched controls. Results demonstrate neutrophil adherence and transmigration and are presented as mean ± SEM from at least three independent experiments.

**Table S1.** Correlation values for genes associated with angiogenesis in tumour areas.

| ***CLEC14A vs.*** | **Spearman r** | **95% CI** | ***P* value**  ***P-*value summary** | |
| --- | --- | --- | --- | --- |
| *LEPR* | −0.42 | −0.58 to −0.21 | <0.0001 | **** |
| *JAG1* | 0.78 | 0.68 to 0.86 | <0.0001 | **** |
| *NRP1* | 0.13 | −0.10 to 0.34 | 0.2469 | ns |
| *NOS3* | 0.82 | 0.73 to 0.88 | <0.0001 | **** |
| *TYMP* | −0.19 | −0.40 to 0.03 | 0.0813 | ns |
| *VEGFB* | 0.53 | 0.35 to 0.67 | <0.0001 | **** |
| *MAPK14* | 0.35 | 0.14 to 0.53 | 0.0011 | ** |
| *ID1* | 0.26 | 0.04 to 0.45 | 0.0172 | * |
| *TNFRSF12A* | 0.28 | 0.06 to 0.47 | 0.0103 | * |
| *ITGAV* | 0.37 | 0.16 to 0.54 | 0.0006 | *** |
| *PRKCA* | 0.62 | 0.46 to 0.74 | <0.0001 | **** |
| *MFGE8* | 0.80 | 0.70 to 0.87 | <0.0001 | **** |
| *SPRY2* | 0.73 | 0.61 to 0.82 | <0.0001 | **** |
| *EFNA1* | −0.74 | −0.83 to −0.62 | <0.0001 | **** |
| *JUN* | −0.02 | −0.24 to 0.20 | 0.8229 | ns |
| *LAMA5* | 0.64 | 0.49 to 0.76 | <0.0001 | **** |
| *TNFSF12* | 0.43 | 0.23 to 0.60 | <0.0001 | **** |
| *VEGFA* | −0.55 | −0.69 to −0.38 | <0.0001 | **** |
| *FGFR2* | 0.49 | 0.31 to 0.65 | <0.0001 | **** |
| *ITGA5* | 0.45 | 0.25 to 0.61 | <0.0001 | **** |
| *GPI* | −0.43 | −0.59 to −0.23 | <0.0001 | **** |
| *RORA* | 0.02 | −0.20 to 0.24 | 0.8439 | ns |
| *FN1* | −0.55 | −0.69 to −0.38 | <0.0001 | **** |
| *DLL4* | 0.67 | 0.53 to 0.77 | <0.0001 | **** |
| *ANGPTL4* | −0.40 | −0.57 to−0.20 | 0.0001 | *** |
| *PTEN* | 0.02 | −0.20 to 0.24 | 0.8585 | ns |
| *MCAM* | 0.77 | 0.66 to 0.85 | <0.0001 | **** |
| *ROBO4* | 0.85 | 0.78 to 0.90 | <0.0001 | **** |
| *SETD2* | 0.59 | 0.43 to 0.72 | <0.0001 | **** |
| *THY1* | 0.53 | 0.35 to 0.67 | <0.0001 | **** |
| *TIE1* | 0.83 | 0.75 to 0.89 | <0.0001 | **** |
| *SHC1* | 0.19 | −0.03 to 0.40 | 0.0808 | ns |
| *CXCR3* | 0.40 | 0.19 to 0.57 | 0.0002 | *** |
| *FGFR1* | 0.85 | 0.78 to 0.90 | <0.0001 | **** |
| *SPRY4* | 0.80 | 0.71 to 0.87 | <0.0001 | **** |
| *CCL2* | 0.69 | 0.55 to 0.79 | <0.0001 | **** |

Genes associated with an angiogenic signature and correlation with *CLEC14A*. Table shows gene symbol, correlation coefficient, and significance value for correlation in tumour-only tissue.

A significant positive correlation is highlighted in pink and a significant negative correlation in blue.

**Table S2.** Correlation values for genes associated with angiogenesis in peri-tumour areas.

| ***CLEC14A* versus** | **Spearman r** | **95% CI** | ***P* value**  **P value summary** | |
| --- | --- | --- | --- | --- |
| *LEPR* | −0.07 | −0.440 to 0.33 | 0.7419 | ns |
| *JAG1* | 0.12 | −0.280 to 0.48 | 0.5528 | ns |
| *NRP1* | −0.27 | −0.590 to 0.12 | 0.1579 | ns |
| *NOS3* | 0.29 | −0.100 to 0.61 | 0.1305 | ns |
| *TYMP* | −0.29 | −0.610 to 0.10 | 0.1327 | ns |
| *VEGFB* | −0.06 | −0.430 to 0.33 | 0.7651 | ns |
| *MAPK14* | 0.02 | −0.370 to 0.40 | 0.9339 | ns |
| *ID1* | −0.12 | −0.480 to 0.28 | 0.5472 | ns |
| *TNFRSF12A* | 0.26 | −0.130 to 0.59 | 0.174 | ns |
| *ITGAV* | −0.26 | −0.580 to 0.14 | 0.1853 | ns |
| *PRKCA* | 0.36 | −0.020 to 0.66 | 0.0565 | ns |
| *MFGE8* | −0.14 | −0.500 to 0.25 | 0.4666 | ns |
| *SPRY2* | 0.03 | −0.360 to 0.40 | 0.8966 | ns |
| *EFNA1* | 0.15 | −0.250 to 0.50 | 0.4496 | ns |
| *JUN* | −0.34 | −0.640 to 0.05 | 0.0733 | ns |
| *LAMA5* | 0.11 | −0.290 to 0.47 | 0.5773 | ns |
| *TNFSF12* | 0.28 | −0.120 to 0.60 | 0.1486 | ns |
| *VEGFA* | −0.27 | −0.590 to 0.13 | 0.1649 | ns |
| *FGFR2* | 0.35 | −0.041 to 0.64 | 0.0699 | ns |
| *ITGA5* | 0.38 | 0.000 to 0.67 | 0.0432 | * |
| *GPI* | −0.46 | −0.720 to −0.09 | 0.0144 | * |
| *RORA* | 0.01 | −0.370 to 0.39 | 0.9493 | ns |
| *FN1* | −0.59 | −0.800 to −0.27 | 0.0009 | *** |
| *DLL4* | 0.44 | 0.065 to 0.70 | 0.02 | * |
| *ANGPTL4* | 0.19 | −0.210 to 0.53 | 0.333 | ns |
| *PTEN* | −0.14 | −0.500 to 0.25 | 0.4683 | ns |
| *MCAM* | 0.48 | 0.120 to 0.73 | 0.01 | * |
| *ROBO4* | 0.43 | 0.058 to 0.70 | 0.0217 | * |
| *SETD2* | 0.24 | −0.150 to 0.57 | 0.2138 | ns |
| *THY1* | −0.11 | −0.480 to 0.28 | 0.5603 | ns |
| *TIE1* | 0.49 | 0.140 to 0.74 | 0.0074 | ** |
| *SHC1* | −0.28 | −0.600 to 0.12 | 0.1545 | ns |
| *CXCR3* | 0.41 | 0.030 to 0.68 | 0.0312 | * |
| *FGFR1* | −0.10 | −0.470 to 0.29 | 0.6101 | ns |
| *SPRY4* | 0.32 | −0.080 to 0.62 | 0.1003 | ns |
| *CCL2* | −0.21 | −0.550 to 0.18 | 0.2754 | ns |

Genes associated with an angiogenic signature and correlation with *CLEC14A*. Table shows Gene ID, correlation coefficient and significance value for correlation in peri-tumour-only tissue. Significant positive correlation is highlighted in pink and significant negative correlation in blue.
